# The cell envelope architecture of Deinococcus: HPI forms the S-layer and SlpA tethers the outer membrane to peptidoglycan

**DOI:** 10.1073/pnas.2305338120

**Published:** 2023-12-12

**Authors:** Tanmay A. M. Bharat, Elitza I. Tocheva, Vikram Alva

**Affiliations:** ^a^Structural Studies Division, MRC Laboratory of Molecular Biology, Cambridge CB2 0QH, United Kingdom; ^b^Department of Microbiology and Immunology, University of British Columbia, Vancouver, BC V6T 1Z3, Canada; ^c^Department of Protein Evolution, Max Planck Institute for Biology Tübingen, Tübingen 72076, Germany

In their recent publication entitled “The structured organization of *Deinococcus radiodurans*’ cell envelope”, Farci et al. (1) present a model for the cell envelope of the bacterium *D. radiodurans*. Despite their commendable cryo-electron microscopy (cryo-EM) images, we believe certain aspects of the proposed model could benefit from further clarification.

Farci et al. (1) propose that the surface layer (S-layer) of *D. radiodurans* is formed by the association of three distinct protein complexes: a type IV-like piliation system (T4P-like), the S-layer deinoxanthin-binding complex (SDBC), and the radial-dimeric (RD) complex, speculated to consist of the hexagonally packed intermediate layer (HPI) protein. They also suggest that these proteins create an ordered array that transcends from the cell surface to the inner membrane, with the localization of HPI remaining unclear. However, these propositions are in stark contrast to the numerous studies from various groups over the past decades, in which room-temperature electron microscopy, cryoelectron crystallography, and atomic force microscopy experiments have consistently identified HPI as the sole S-layer protein (2–7).

**Fig. 1.**
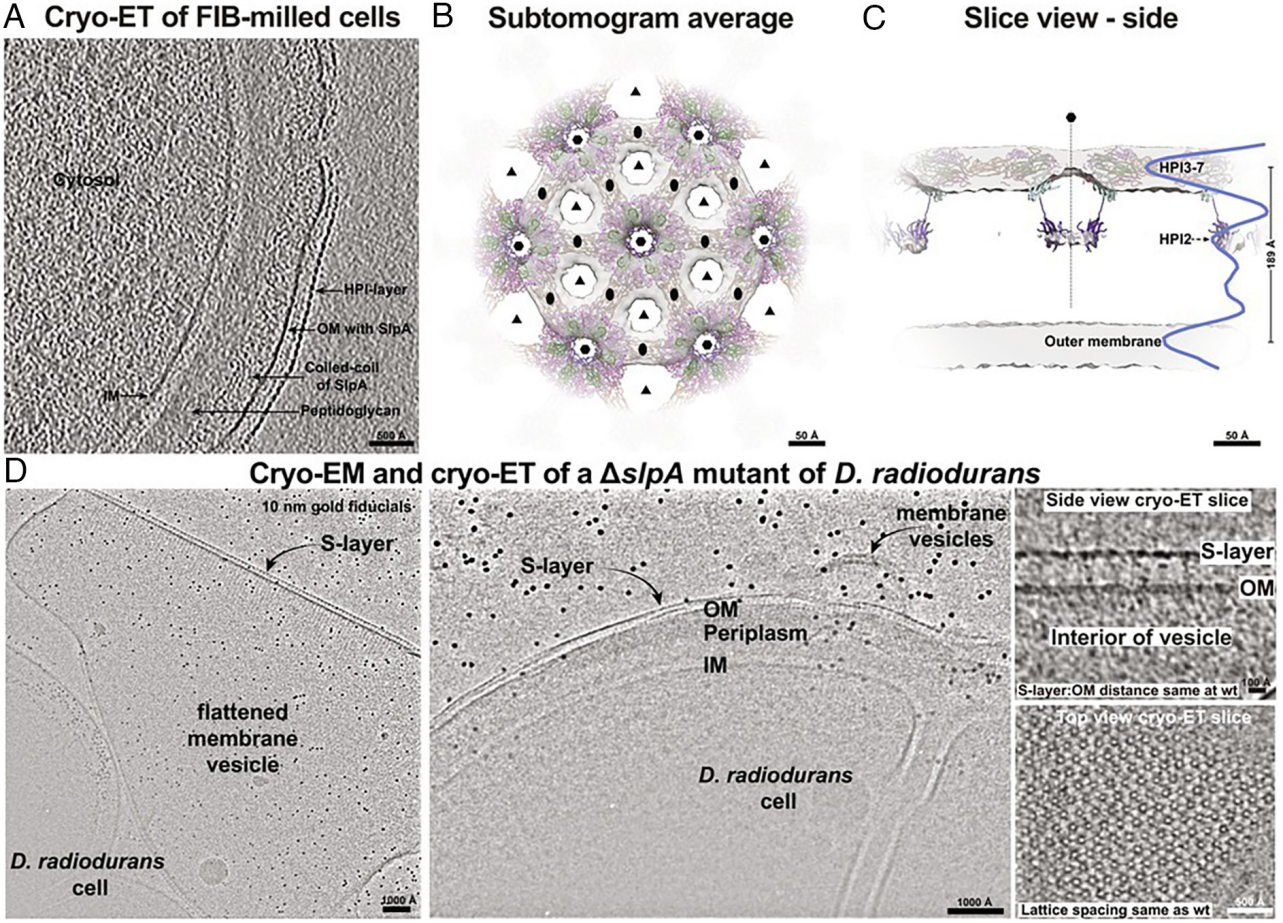
(*A*) FIB milling and cryo-ET of *D. radiodurans* cells shows periodicity only in the S-layer. (*B* and *C*) Subtomogram average of the S-layer, shown in two orthogonal orientations confirms that the density corresponds only to the HPI protein (structure fitted into map, see ref. 8). (*D*) Two-dimensional cryo-EM images (*Left* and *Middle*) of the Δ*slpA* mutant revealed the presence of OM vesicles, likely due to OM dissociation from the PG (10), whereas cryo-ET (*Right*) of the vesicles revealed that the morphology and ultrastructure of the S-layer assembled on vesicular membranes remained unaffected.

In our recent work, we determined a 2.5-Å-resolution cryo-EM structure of the S-layer, revealing that HPI forms a hexagonal lattice (8). Utilizing cryo-focused ion beam (FIB) milling and cryo-electron tomography (cryo-ET), we directly imaged plunge-frozen *D. radiodurans* cells in near-native conditions and confirmed that the subtomogram average of the S-layer corresponds to our cryo-EM structure (Fig. 1 *A*–*C*), further supporting the model that HPI is the sole component of the S-layer (8, 9). Notably, the densities Farci et al. interpret as the RD and T4P-like complexes correspond to the HPI lattice.

**Fig. 2.**
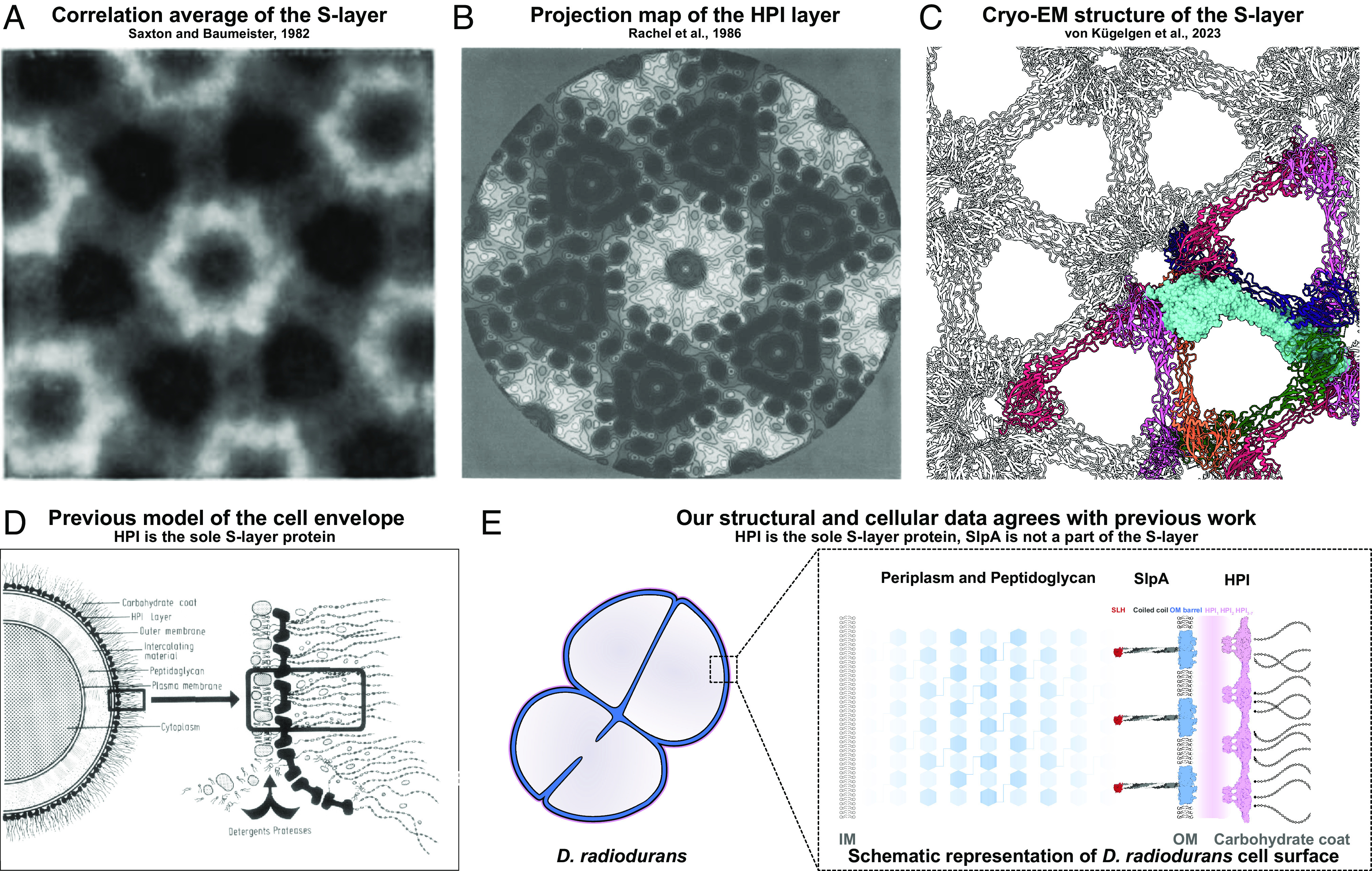
(*A*) Correlation averaging of the *D. radiodurans* S-layer shows the characteristic hexagonal pattern (6). (*B*) Projection map of the lattice confirms that the S-layer is composed of the HPI protein (3). (*C*) Our atomic structure of the HPI lattice confirms previous averaging data and shows that HPI is the sole S-layer protein (8). (*D*) Model of the *D. radiodurans* proposed by ref. 2, which agrees with data from several labs (2–10). (*E*) Our model based on atomic structural data and data from FIB-milled cells agrees with the previous model (8, 9).

Furthermore, Farci et al. (1) propose that the SDBC complex, which includes the protein SlpA, arranges itself in a tiled pattern in the *D. radiodurans* outer membrane (OM) and associates with the S-layer, conforming to its symmetry. Contrary to this, our cryo-EM results indicate that SlpA does not bind stoichiometrically to the S-layer. This finding is further supported by our cryo-EM and cryo-ET imaging of the Δ*slpA* mutant, which showed no discernible differences in the S-layer compared to the wild-type (Fig. 1*D*) (10). Additionally, SlpA’s biological role is to tether the OM to the peptidoglycan layer (Fig. 2), maintaining the integrity of the OM (10). It assembles into homotrimers, with its C-terminal domain forming 30-stranded β-barrels in the OM and its central segment forming coiled-coil stalks that connect the OM to the peptidoglycan layer via N-terminal S-layer homology domains (10). Lastly, our data did not provide any evidence of regular tiling of SlpA in conjunction with a T4P-like protein in the OM of *D. radiodurans*, which followed the HPI S-layer symmetry (8).

In summary, our results (Figs. 1 and 2), together with previous studies (2–10), do not support Farci et al.’s envelope model (1). We believe that it is necessary to constructively highlight this discrepancy, given its significant implications for our understanding of bacterial cell envelope architecture and the evolution of early-branching bacterial phyla. We anticipate that future high-resolution in situ structures will further refine our *D. radiodurans* cell envelope model.
